# *APOL1* and chronic kidney disease in pediatrics: a study from the Biorepository and Integrative Genomics Initiative

**DOI:** 10.1007/s00467-026-07385-5

**Published:** 2026-06-12

**Authors:** Rima S. Zahr, Lokesh Chinthala, Akram Mohammed, Csaba P. Kovesdy, Robert L. Davis

**Affiliations:** 1 Department of Pediatric Nephrology and Hypertension, University of Tennessee Health Science Center, Memphis, USA; 2 Department of Center for Biomedical Informatics, University of Tennessee Health Science Center, Memphis, USA; 3 Department of Nephrology, University of Tennessee Health Science Center, Memphis, USA

**Keywords:** Pediatrics, Albuminuria, CKD, *APOL1*, Genomics

## Abstract

**Background:**

*APOL1* variants confer increased risk for kidney disease in individuals of African ancestry, particularly in homozygous or compound heterozygous states. A missense variant, p.N264K, has been shown to attenuate this risk when co-inherited with the G2 allele. While these associations are established in adults, data in pediatric populations remain limited. Using the University of Tennessee Health Science Biorepository and Integrative Genomics initiative, we assessed chronic kidney disease (CKD) risk by *APOL1* high-risk genotype and the potential protective effect of p.N264K in a diverse pediatric cohort.

**Methods:**

This is a case–control study of African American children enrolled in the BIG initiative at Le Bonheur Children’s Hospital (2014–2022). Composite CKD was defined by eGFR < 90 mL/min/1.73 m^2^ or albuminuria (UACR > 30 mg/g) on two occasions ≥ 90 days apart. The p.N264K variant status was assessed in patients with *APOL1* high-risk (HR). Logistic regression models adjusted for age, sex, and five principal components of ancestry assessed associations with CKD and albuminuria.

**Results:**

CKD cases had 8% higher odds of *APOL1* HR status (Odds Ratio (OR): 1.08, 95% Confidence Interval (CI): 0.88–1.33). Stronger associations were observed in cases with albuminuria and *APOL1* HR status (OR: 1.41, 95% CI: 1.02–1.92, p = 0.03). The p.N264K variant was detected in 4.3% of cases and 4.5% of controls, and appeared to attenuate CKD risk among HR individuals, though sample size limited statistical power.

**Conclusions:**

*APOL1* HR genotypes were associated with albuminuria in African American children. The p.N264K variant may modify this risk, warranting further study.

**Graphical abstract:**

**Figure:**
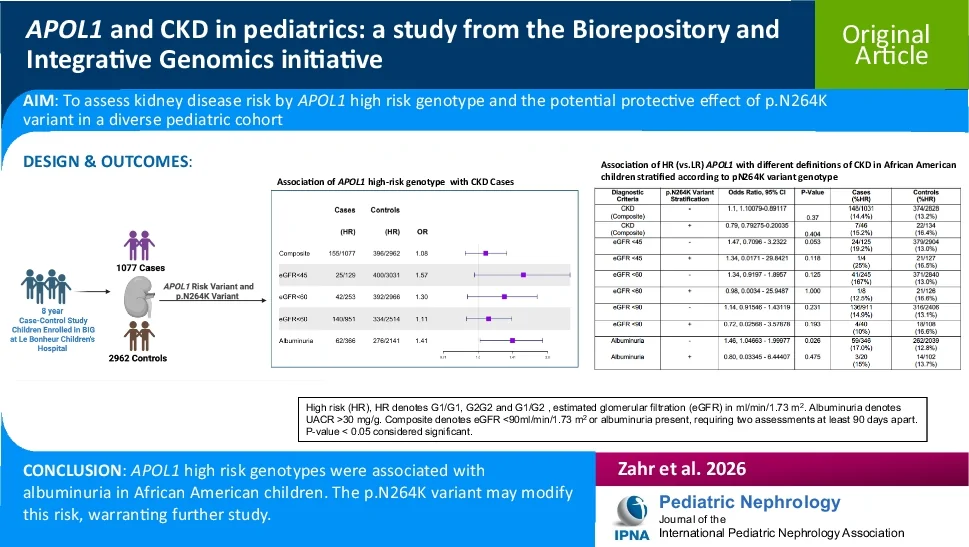
A higher resolution version of the Graphical abstract is available as Supplementary information

**Supplementary Information:**

The online version contains supplementary material available at https://doi.org/10.1007/s00467-026-07385-5.

## Introduction

Individuals of African ancestry carrying coding variants in apolipoprotein L1 gene (*APOL1*), G1 and G2, are at increased risk for a spectrum of kidney diseases [1, 2]. These variants confer resistance against trypanosome infections that are endemic to sub-Saharan Africa and explain their high frequency in individuals with African ancestry [3, 4]. These *APOL1* G1 and G2 variants, in either homozygous (G1/G2 or G2/G2) or compound heterozygous (G1/G2) forms have been associated with poorer kidney function and hypertension in Africans, South Africans and African Americans [3, 5–9]. Recent research has identified a rare missense variant in *APOL1*, p.N264K (chr22:35265628 C > A; rs73885316), (< 1–2% allele frequency) which appears to mitigate kidney disease risk associated with the high risk G2 allele of *APOL1* gene when co-inherited [10–13]. Functional genetic studies in cell models have demonstrated that the *p.N264K* variant blocked APOL1 pore-forming function and ion channel conduction and reduced toxicity of *APOL1* HR mutations [13]. These studies have independently demonstrated that this variant is able to reduce the risk of *APOL1* G2-mediated focal segmental glomerulosclerosis (FSGS) and kidney disease to almost the same level as the traditional low risk genotypes [13, 14].

Growing data support a role for *APOL1* HR genotypes as modifiers of kidney disease severity in children, though the effects appears to vary by underlying condition. One study from the Chronic Kidney Disease in Children (CKiD) and Nephrotic Syndrome (NEPTUNE) cohorts demonstrated that children with APOL*1* HR genotypes exhibited more aggressive disease progression and higher prevalence of focal segmental glomerulosclerosis [15]. Additionally, children with sickle cell disease (SCD) who carry *APOL1* HR develop albuminuria earlier than their SCD counterparts without HR genotypes [16]. Interestingly, in a recent study from Glomerulonephritis Cohort (CureGN), adults born preterm (< 37 weeks gestation) were more likely to have *APOL1* HR. However, a pilot study examining children and adults with congenital anomalies of the kidney and urinary tract (CAKUT) failed to demonstrate an association between *APOL1* and kidney outcomes (hypertension, proteinuria or CKD) [17]. While the impact of these variants on kidney health has been well studied in adults, fewer studies have assessed the relationship of the *APOL1* high-risk (HR) variants (G1/G1, G2/G2, G1/G2) in the context the p.N264K variant with kidney disease risk in the pediatric population.

To further expand our understanding of how *APOL1* variants affect kidney disease development in children, we utilized genomic data from the University of Tennessee Health Science Center (UTHSC) Biorepository and Integrative Genomics (BIG) initiative which links comprehensive electronic health records from consenting pediatric patients cared for at Le Bonheur Children’s Hospital in Memphis, TN [18]. Memphis is historically a disadvantaged US metropolitan area where the majority of residents are African Americans with a high overall poverty rate and health disparities found in children in this area [19–21]. A recent study from BIG demonstrated a high percent of non-European ancestry with high proportion of those with African ancestry [22].

In our study, we aimed to 1) describe the baseline characteristics of children within the BIG initiative by *APOL1* HR genotype, 2) assess risk of chronic kidney disease (CKD) by *APOL1* HR genotype, and 3) characterize the protective effect of p.N264K variant among children with the *APOL1* HR genotypes.

## Methods

This is a case–control study of African American children cared for at Le Bonheur Children’s Hospital (LBCH) in Memphis, TN. Pediatric patients consented in the BIG initiative at UTHSC and LBCH were studied. LBCH is the primary pediatric care center in Memphis. Recruitment into BIG was launched in October 2015 and spans inpatient rooms, intensive care units, outpatient clinics and the emergency department. This study was approved by the UTHSC Institutional Review Board, protocol 23–09622-XP.

Clinical data from both inpatient and outpatient visits were obtained utilizing the electronic medical record from the enterprise data warehouse (EDW) at UTHSC between 2014 and 2022. Cases and controls were identified using available laboratory data from the patient record to include urine albumin–creatinine ratio (UACR), urine protein–creatinine ratio (UPCR), urine dipstick results, and serum creatinine. We converted UPCR and urine dipstick protein to UACR values using the previously published conversion equation [23] and categorized the resulting UACR values as < 30 mg/g and > 30 mg/g. Cases were defined as having composite CKD based on one of two criteria, each requiring two assessments conducted at least 90 days apart [24]: 1) eGFR < 90 mL/min/1.73 m^2^ (calculated using the Chronic Kidney Disease in Children Under 25 formula (CKiD U25), creatinine only equation) [25, 26]; or 2) albuminuria, UACR > 30 mg/g. Children who did not fulfill the case criteria were included as controls. Sensitivity analyses were performed with cutoffs of eGFR < 60 and < 45 ml/min/1.73 m^2^. Patient age and body mass index (BMI) were obtained from the time point closest to first positive lab test (urine test or serum creatinine) for cases and first negative lab test for controls. Diagnosis of hypertension (HTN) was obtained from the patient record using ICD 9/10 codes for HTN. Patient race/ethnicity identification was self-reported and obtained from the medical record.

### Genotyping

We utilized DNA sequencing data from the Le Bonheur Children’s Hospital and UTHSC Biorepository and Integrative Genomics (BIG) database from over 13,000 patients. Data was generated by Regeneron Genetics on Illumina NovaSeq6000 using 75 bp paired-end sequencing. All whole-exome sequences and genotyping-by-sequencing data were aligned to the GRCh38 reference genome with BWA-MEM. Variant calling was conducted using DeepVariant with a custom exome model, and variants were annotated using the Variant Effect Predictor (VEP 110) and gnomAD v3 population database.

From the sequencing data, participants were categorized with zero (G0/G0), one (G1/G0) or two (G1/G1, G2/G2, G1/G2) risk alleles. We further combined the zero and one groups together as low-risk (LR) and two risk alleles as high-risk (HR). The *APOL1* p.N264K variant status was also assessed.

### Statistical methods

To assess distributions in participant demographic and clinical characteristics between two groups, Mann–Whitney U test was used for continuous variables, and chi-square test was used for categorical variables. A generalized linear model (GLM) with a binomial distribution and a logit link function was fitted to the data, equivalent to performing logistic regression [27]. The coefficients from the logistic regression were exponentiated to calculate the odds ratios (OR), and 95% confidence intervals for CKD risk. Models were adjusted for age (modeled with linear and cubic terms), sex, and population structure using five principal ancestry components (PCs) as determined by the elbow point of the scree plot. Due to the considerable evidence that exists in the medical literature describing the interaction between *APOL1* HR Status and p.N264K variant status, we present our results stratified by p.N264K variant status. P < 0.05 was considered significant.

## Results

### General description of patient characteristics

**Fig. 1:**
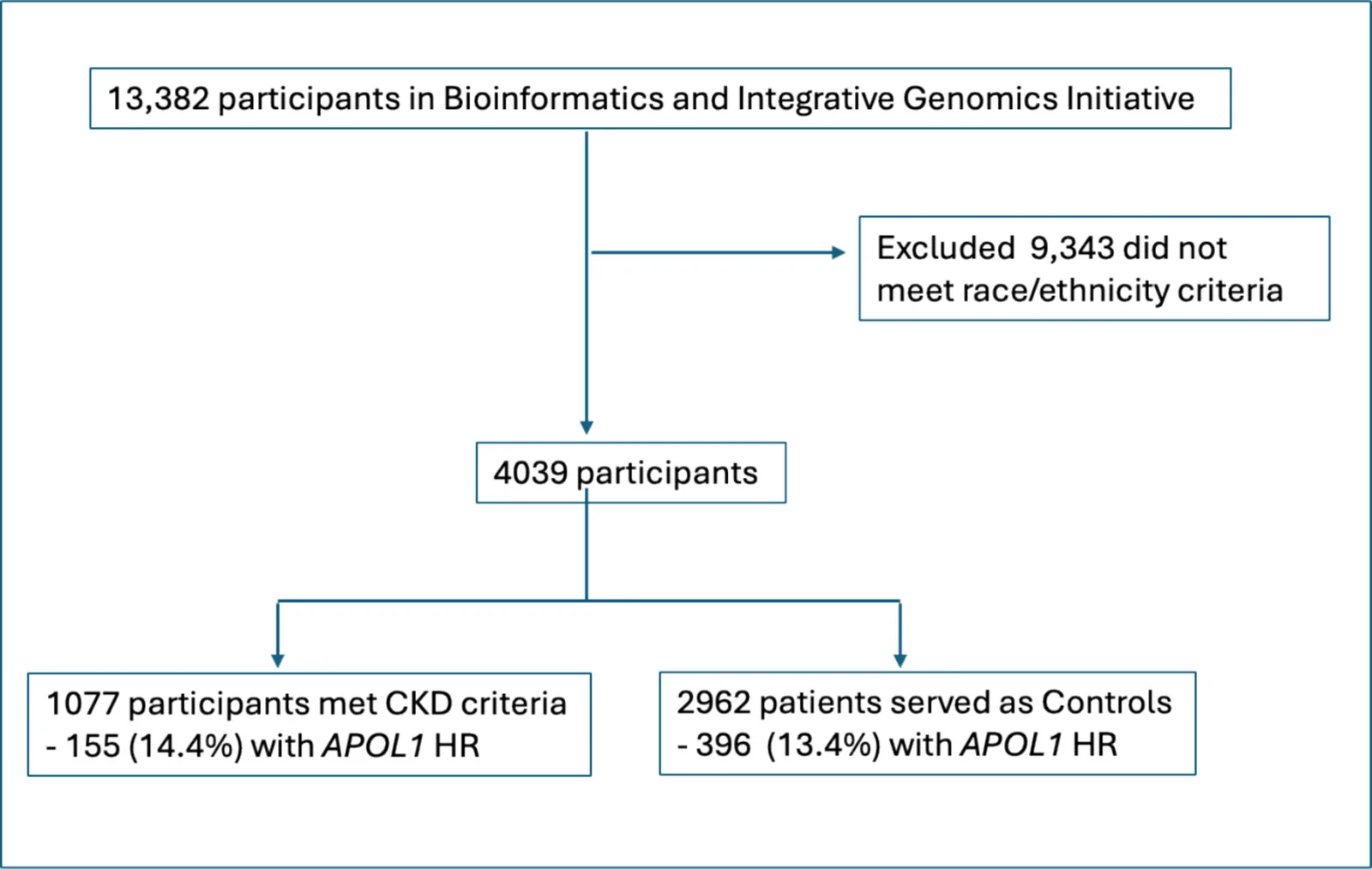
Flow chart of study analysis

Of the 13,382 participants in the BIG, 4,039 were African American. From this group, 1077 children met case definition for CKD, and 2962 children were controls, Fig. 1. Children that met the case definition were older, (6.1 years vs. 4.5 years), had a lower mean eGFR, and higher proportion of hypertension, 11.8% vs. 0.9%, p < 0.001, Table 1. Among CKD cases, 14.4% (155/1077) had an *APOL1* HR variant, compared to 13.4% (396/2962) in controls. The p.N264K variant was detected in 4.3% (46/1077) of cases and 4.5% (134/2962) of controls. Descriptive findings between HR vs. LR *APOL1* status can be found in Supplementary Table 1.

**Table 1:** Baseline characteristics of pediatric patients by case–control status

Parameter	Case (N = 1,077)	Control (N = 2,962)	p-value
Male	491 [45.6%]	1,602 [54.1%]	< 0.001
Age	6.1 [0.2–14.3]	4.5 [0.9–11.4]	0.35
BMI	17.9 [15.2–23.2]	17.0 [14.9–20.7]	< 0.001
UACR	39.5 [12.0–67.0]	12.0 [12.0–25.0]	< 0.001
Albuminuria (UACR > 30 mg/g)	378 (35.1%)	0 (0.0%)	-
eGFR (ml/min/1.73 m^2^)	86.8 [70.5–101.2]	108.2 [93.1–124.7]	< 0.001
eGFR < 45 ml/min/1.73 m^2^	129 (12.0%)	0 (0.0%)	-
eGFR < 60 ml/min/1.73 m^2^	253 (23.5%)	0 (0.0%)	-
eGFR < 90 ml/min/1.73 m^2^	951 (88.3%)	0 (0.0%)	-
Diagnosis of HTN	127 (11.8%)	27 (0.9%)	< 0.001

Median and Interquartile Range (IQR) displayed for continuous variables and count and frequency displayed for categorical variables. Albuminuria is a categorical variable (yes/no: UACR > 30 mg/g). UACR was assessed as a continuous variable. “- “ p-value not reported due to 0% prevalence in control group

### Association between APOL1 and CKD

CKD cases (composite definition) had 8% higher odds of *APOL1* HR status, (Odds Ratio (OR): 1.08, 95% Confidence Interval (CI): 0.88–1.33), Table 2. The association between cases and *APOL1* HR status was stronger when using eGFR cutoffs of < 60 and < 45 ml/min/1.73 m^2^ (OR: 1.30, 95% CI: 0.90–1.83, and OR: 1.57, 95% CI: 0.97–2.44, respectively), Table 2. Albuminuria cases had 41% higher odds of *APOL1* HR status (OR: 1.41 95% CI: 1.02–1.92, p = 0.032), Table 2. Supplemental Table 2 presents the association between cases and *APOL1* HR status across three diagnostic definitions for albuminuria.

**Table 2:** Association of *APOL1* high-risk genotype (vs. low risk) with CKD

CKD by various diagnostic criteria	Odds Ratio, 95% CI	P-value	Cases (%HR)	Controls (%HR)
CKD (Composite)	1.08, 0.88–1.33	0.44	155/1077 (14.4%)	396/2962 (13.4%)
eGFR < 45 ml/min/1.73 m^2^	1.57, 0.964–2.4643	0.053	25/129 (19.3%)	400/3031 (13.1%)
eGFR < 60 ml/min/1.73 m^2^	1.30, 0.90–1.83	0.156	42/253 (16.6%)	392/2966 (13.2%)
eGFR < 90 ml/min/1.73 m^2^	1.11, 0.89–1.38	0.349	140/951 (14.7%)	334/2514 (13.2%)
Albuminuria	1.41, 1.02–1.92	0.032	62/366 (16.9%)	276/2141 (12.8%)

^*^High risk (HR), HR denotes G1/G1, G2G2 and G1/G2, otherwise LR, estimated glomerular filtration (eGFR) in ml/min/1.73 m^2^. Composite denotes eGFR < 90 ml/min/1.73 m^2^ or albuminuria present, requiring two assessments at least 90 days apart. Albuminuria denotes UACR > 30 mg/g. Odds ratio was determined by logistic regression model and adjusted for age, sex, and five principal components of ancestry. P-value < 0.05 considered significant

Table 3 presents *APOL1* HR results stratified by p.N264K variant status (presence vs. absence). The relationship between *APOL1* HR status and cases (CKD composite) was lower in the presence of p.N264K positive than when it was p.N264K negative (OR: 0.94, 95% CI: 0.2047–2.9809 vs. (OR: 1.22, 95% CI: 0.9445–1.5488, respectively). At both eGFR < 45 and < 60, the odds were lower with p.N264K positive vs. those with p.N264K variant negative for both eGFR cutoffs (for eGFR < 45: OR: 1.34, 95% CI: 0.017–29.8 vs. OR: 1.47, 95% CI: 0.71–3.23; and for eGFR < 60: OR: 0.97, 95% CI: 0.003–25.9 vs. OR:1.33, 95% CI: 0.919–1.89, respectively). Using cases defined by albuminuria, there was also a lower OR when N264K positive status (OR: 0.799, 95% CI: 0.033- 6.44, p = 0.47) vs. p.N264K negative (OR: 1.45, 95% CI: 1.04–1.99, p = 0.03). CKD outcomes by G1 or G2 allele status stratified by p.N264K variant status are provided in Supplementary Table 3.

**Table 3:** Association of HR (vs. LR) *APOL1* with different definitions of CKD in African American children stratified according to pN264K variant genotype

CKD by various diagnostic criteria	p.N264K variant stratification	Odds ratio, 95% CI	P-Value	Cases (%HR)	Controls (%HR)
CKD (Composite)	-	1.1, 1.10079–0.89117	0.37	148/1031 (14.4%)	374/2828 (13.2%)
CKD (Composite)	+	0.79, 0.79275–0.20035	0.404	7/46 (15.2%)	22/134 (16.4%)
eGFR < 45	-	1.47, 0.7096–3.2322	0.053	24/125 (19.2%)	379/2904 (13.0%)
eGFR < 45	+	1.34, 0.0171–29.8421	0.118	1/4 (25%)	21/127 (16.5%)
eGFR < 60	-	1.34, 0.9197–1.8957	0.125	41/245 (167%)	371/2840 (13.0%)
eGFR < 60	+	0.98, 0.0034–25.9487	1.000	1/8 (12.5%)	21/126 (16.6%)
eGFR < 90	-	1.14, 0.91546–1.43119	0.231	136/911 (14.9%)	316/2406 (13.1%)
eGFR < 90	+	0.72, 0.02568–3.57878	0.193	4/40 (10%)	18/108 (16.6%)
Albuminuria	-	1.46, 1.04663–1.99977	0.026	59/346 (17.0%)	262/2039 (12.8%)
Albuminuria	+	0.80, 0.03345–6.44407	0.475	3/20 (15%)	14/102 (13.7%)

^*^High risk (HR), HR denotes G1/G1, G2G2 and G1/G2, otherwise LR, estimated glomerular filtration (eGFR) in ml/min/1.73 m^2^. Albuminuria denotes UACR > 30 mg/g. Odds ratio was determined by logistic regression model and adjusted for age, sex, and five principal components of ancestry. P-value < 0.05 considered significant

## Discussion

In this study, we investigated a large, pediatric cohort to identify the association of *APOL1* risk genotypes with CKD. By leveraging data from the Biorepository and Integrative Genomics Initiative, we were able to conduct a robust genotype–phenotype analysis. Our findings demonstrate that the *APOL1* HR genotype is associated with CKD. The strength of this association was slightly attenuated in the presence of the p.N264K variant, although the low number of individuals in subgroups may have precluded the detection of statistically significant differences. In addition, the associations we found in our study are generally lower than observed in adults. This may reflect the developmental timing of *APOL1-*mediated disease and the heterogeneity of CKD in childhood, which differs from adult-onset CKD. Despite these considerations, our findings provide further evidence that *APOL1* high-risk genotypes contribute to increased risk for CKD in African American children.

*APOL1* risk variants are well-established contributors to the disproportionate burden of CKD among individuals of African ancestry. In Memphis, TN, a region with a large African American population, the impact of these genetic variants is especially relevant. In our study, we found a large proportion (27%) of children with CKD, but this high proportion reflects the referral nature of the patient population. Understanding the role of *APOL1* in contributing to CKD in the pediatric populations is critical, as early identification of at-risk individuals could inform targeted screening, guide clinical management, and ultimately improve long-term kidney outcomes [28]. Moreover, integrating genetic risk assessment into public health strategies may help address existing health disparities and promote equity in kidney disease prevention and care [8]. However, it is important to note that the BIG cohort provides genomic characterization of children with rare and complex conditions, rather than a representative screening population. Current guidelines discourage routine *APOL1* testing in children except in narrowly defined contexts [29].

In this study, we faced limitations common to retrospective studies, including lack of uniform CKD measures. Some participants had urinary markers (e.g., UACR or urine dipsticks) as CKD indicators, while others had only serum creatinine levels available, potentially impacting the consistency of CKD classification across the cohort. This variability could introduce bias or limit the comparability of CKD diagnoses. In addition, due to the small number of p.N264K carriers we were unable to carry out conclusive examinations of G1/G2 and G2/G2 in the setting of p.N264K variant. Previous studies have reported a significant interaction between the p.N264K variant and *APOL1* HR [13]. These studies benefited from larger adult cohorts of patients with CKD, whereas our sample size though large was underpowered to detect this interaction. Future larger studies of *APOL1* and of p.N264K carriers will be necessary to further fully study its effects on CKD progression.

Despite the limitations of our study, one of its key strengths is the use of a large, well genotyped pediatric cohort linked to clinical electronic health record data, allowing for an in-depth analysis of genotype–phenotype relationship. This linkage is particularly valuable for studying CKD in a population which is historically underrepresented in genetic research. Despite the small number of children with the p.N264K variant in the setting of our relatively large sample size, we observed a lower odds ratio of CKD (eGFR < 60 and < 45) in *APOL1* HR children in the presence of p.N264K.

## Conclusion

Our study highlights the significance of *APOL1* HR variants in CKD prevalence among African American children. Future research can help develop strategies for early identification and targeted interventions in at-risk pediatric populations.

## Supplementary Information

Below is the link to the electronic supplementary material.

ESM 1 (DOCX 24.3 KB)

ESM 2 Graphical abstract (PPTX 421 KB)

## Data Availability

The data supporting this study’s findings are sourced from the Biorepository and Integrative Genomics (BIG) and are not publicly available due to privacy and ethical restrictions. Access to the data is restricted to protect participant confidentiality and comply with institutional and regulatory requirements. Researchers may request access to the data after obtaining approval from the University of Tennessee Health Science Center (UTHSC) Institutional Review Board (IRB) and the BIG Research Oversight Committee. Requests should be submitted via the BIG portal at https://uthsc.edu/cbmi/big/. For further assistance, please contact the authors.
