# Supplementary material for: APOL1 and chronic kidney disease in pediatrics: a study from the Biorepository and Integrative Genomics Initiative
Source: Pediatr Nephrol. 2026 Jun 12;41(11):4057–63. doi: 10.1007/s00467-026-07385-5 (PMC13612674; doi:10.1007/s00467-026-07385-5)
Supplement: Supplementary file 1 [file 467_2026_7385_MOESM1_ESM.docx]

**Supplemental Material**

*APOL1* and CKD in Pediatrics: A Study from the Biorepository and Integrative Genomics Initiative

Authors: Rima S. Zahr^1^ (X handle: @drRimaZahr), Lokesh Chinthala^2^, Akram Mohammed^2^, Csaba P. Kovesdy^3^, Robert L. Davis^2^

UTHSC Department of ^1^Pediatric Nephrology and Hypertension, ^2^Center for Biomedical Informatics and ^3^Nephrology

**Supplementary Table 1. Descriptive Characteristics of Pediatric Patients with HR vs. LR *APOL1* status**

| **Parameter** | **HR**  **(N= 551)** | **LR**  **(N= 3,488)** | **p-value** |
| --- | --- | --- | --- |
| Male | 287 (52.1%) | 1,806 (51.8%) | 0.93 |
| Age | 4.7 [0.8-12.4] | 4.7 [0.7-12.2] | 1.00 |
| BMI | 16.9 [14.9-21.7] | 17.2 [14.9, 21.4] | 0.57 |
| Albuminuria (>30 mg/g) | 62 (11.3%) | 316 (9.1%) | 0.12 |
| UACR (mg/g) | 12.0 [12.0-67.0] | 12.0 [12.0-59.3] | 0.04 |
| eGFR (ml/min/1.73m2) | 101.2 [82.1-118.8] | 102.5 [86.4-120.0] | 0.06 |
| CKD  (eGFR < 45 ml/min/1.73m^2^) | 25 (4.5%) | 104 (3.0%) | 0.07 |
| CKD  (eGFR < 60 ml/min/1.73m^2^) | 42 (7.6%) | 211 (6.0%) | 0.19 |
| CKD  (eGFR < 90 ml/min/1.73m^2^) | 140 (25.4%) | 811 (23.3%) | 0.29 |
| Diagnosis of HTN | 33 (6.0%) | 121 (3.5%) | 0.01 |

*Age and BMI taken to the closest lab time point, Median and Interquartile Range (IQR) displayed for continuous variables and count and frequency displayed for categorical variables. High Risk *APOL1* defined as G1/G1, G1/G2 or G2/2, otherwise low risk. Albuminuria denotes patients with UACR>30mg/g. P-value <0.05 considered significant

**Supplemental Table 2. Association of APOL1 high-risk genotype (vs. low) with Albuminuria**

| **CKD by various diagnostic criteria** | **Odds Ratio, 95% CI** | **P-value** | **Cases**  **(%HR)** | **Controls**  **(%HR)** |
| --- | --- | --- | --- | --- |
| Albuminuria (Composite) | 1.41, 1.02-1.92 | 0.032 | 62/366  (16.9%) | 276/2141  (12.8%) |
| UACR_Albuminuria | 2.43, 1.33-4.53 | 0.004 | 37/122  (30.3%) | 23/128  (18.0%) |
| Dipstick_Albuminuria | 1.36, 0.95-1.91 | 0.081 | 49/256  (19.2%) | 268/1832  (14.6%) |

*High risk (HR), HR denotes G1/G1, G2G2 and G1/G2 otherwise LR. Composite Albuminuria denotes UACR >30mg/g (UACR with converted from UPCR and urine dipstick). Odds ratio was determined by logistic regression model and adjusted for age, sex, and five principal components of ancestry. P-value < 0.05 considered significant.

**Supplemental Table 3.** Association of *APOL1* High-Risk Genotypes (HR1:G1/G1, HR2: G1/G2 and G2/G2) with CKD Definitions in Children Stratified by N264K Variant Status

| **CKD by Various Criteria** | **Stratification** | **Genotype** | **Odds Ratio** | **95% CI Lower** | **95% CI Upper** | **P-Value** | **Cases HR1** | **Controls**  **HR1** | **Cases**  **HR2** | **Controls**  **HR2** | **Cases**  **LR** | **Controls**  **LR** |
| --- | --- | --- | --- | --- | --- | --- | --- | --- | --- | --- | --- | --- |
| CKD  (Composite) | N264K_status: - | HR1 | 1.3033 | 0.96418 | 1.74605 | 0.08428 | 70 | 154 | - | - | 883 | 2454 |
| CKD  (Composite) | N264K_status: - | HR2 | 1.07179 | 0.76554 | 1.47215 | 0.67896 | - | - | 78 | 220 | 883 | 2454 |
| CKD  (Composite) | N264K_status: + | HR1 | NA | NA | NA | NA | 0 | 0 | - | - | 39 | 112 |
| CKD  (Composite) | N264K_status: + | HR2 | 0.93543 | 0.20471 | 2.98086 | 0.84781 | - | - | 7 | 22 | 39 | 112 |
| eGFR<45 | N264K_status: - | HR1 | 1.86544 | 0.75444 | 4.73450 | 0.01006 | 13 | 162 | - | - | 101 | 2525 |
| eGFR<45 | N264K_status: - | HR2 | 1.23621 | 0.42771 | 3.09772 | 0.65384 | - | - | 11 | 217 | 101 | 2525 |
| eGFR<45 | N264K_status: + | HR1 | NA | NA | NA | NA | 0 | 0 | - | - | 3 | 106 |
| eGFR<45 | N264K_status: + | HR2 | 1.34280 | 0.01708 | 29.84213 | 0.11812 | - | - | 1 | 21 | 3 | 106 |
| eGFR<60 | N264K_status: - | HR1 | 1.21307 | 0.68596 | 2.01541 | 0.48884 | 16 | 162 | - | - | 204 | 2469 |
| eGFR<60 | N264K_status: - | HR2 | 1.45676 | 0.91001 | 2.24286 | 0.11364 | - | - | 25 | 209 | 204 | 2469 |
| eGFR<60 | N264K_status: + | HR1 | NA | NA | NA | NA | 0 | 0 | - | - | 7 | 105 |
| eGFR<60 | N264K_status: + | HR2 | 0.97895 | 0.00340 | 25.94865 | 1.00000 | - | - | 1 | 21 | 7 | 105 |
| eGFR<90 | N264K_status: - | HR1 | 1.35279 | 0.98124 | 1.84872 | 0.06487 | 65 | 131 | - | - | 775 | 2090 |
| eGFR<90 | N264K_status: - | HR2 | 1.00838 | 0.74810 | 1.34568 | 0.95554 | - | - | 71 | 185 | 775 | 2090 |
| eGFR<90 | N264K_status: + | HR1 | NA | NA | NA | NA | 0 | 0 | - | - | 36 | 90 |
| eGFR<90 | N264K_status: + | HR2 | 0.71464 | 0.02568 | 3.57878 | 0.19398 | - | - | 4 | 18 | 36 | 90 |
| Albuminuria | N264K_status: - | HR1 | 1.43180 | 0.87317 | 2.26946 | 0.15037 | 24 | 112 | - | - | 287 | 1777 |
| Albuminuria | N264K_status: - | HR2 | 1.48686 | 0.97900 | 2.20965 | 0.06242 | - | - | 35 | 150 | 287 | 1777 |
| Albuminuria | N264K_status: + | HR1 | NA | NA | NA | NA | 0 | 0 | - | - | 17 | 88 |
| Albuminuria | N264K_status: + | HR2 | 0.79903 | 0.03345 | 6.44407 | 0.47560 | - | - | 3 | 14 | 17 | 88 |

*High risk (HR), HR1: G1/G1 and HR2: G1/G2 and G2/G2 otherwise LR, estimated glomerular filtration (eGFR) in ml/min/1.73m^2^. Albuminuria denotes UACR >30mg/g. Odds ratio was determined by logistic regression model and adjusted for age, sex, and five principal components of ancestry. P-value < 0.05 considered significant.
